# Antibiotic-induced morphological changes enhance phage predation

**DOI:** 10.1371/journal.ppat.1013546

**Published:** 2025-10-03

**Authors:** Julián Bulssico, Swapnesh Panigrahi, Mélanie Matveeva, Nicolas Ginet, Mireille Ansaldi

**Affiliations:** Laboratoire de Chimie Bactérienne, UMR7283, Institut de Microbiologie de la Méditerranée, Centre National de la Recherche Scientifique, Aix-Marseille Université, Marseille, France; University of Colorado Anschutz Medical Campus, UNITED STATES OF AMERICA

## Abstract

Due to the high public health risk posed by antibioresistance, phage therapy - the use of bacteriophages as antibacterial agents - is experiencing renewed interest. As the combined administration of antibiotics and phages is a common practice in compassionate treatments, our research focuses on the effects of antibiotics on phage predation, which may be of crucial importance for phage therapeutic applications. A distinctive manifestation of phage infection in solid media is the appearance of lysis plaques, corresponding to the circular thinning of a bacterial lawn. During plaque formation, successive cycles of phage replication take place from a single point of infection and spread radially in a lawn of immobilized bacterial hosts. Many factors affect plaque size, such as the composition and the reticulation of the propagation matrix, the characteristics of the phage, but also parameters related to the physiology of the bacterial host. It has also been known for decades that some antibiotics enable phages to spread more rapidly, resulting in better bacterial eradication. This phenomenon, called Phage-Antibiotic Synergy (PAS), is evidenced by larger lysis plaques on solid media. Our previous experimental work has focused on the phage characteristics and pointed to enhanced adsorption as a key factor leading to more efficient predation. However, since sublethal antibiotic concentrations can drastically affect bacterial physiology - for instance halting cell division in the case of ciprofloxacin or ceftazidime - and since plaque formation is strongly influenced by host growth dynamics, a comprehensive model integrating both the host growth and phage infection parameters is required to investigate PAS. We characterized the epidemics of two different phages (T5 and T7) during *E. coli* MG1655 infection on semi-solid media in the presence of sublethal antibiotic concentrations that affect (or not) cell morphology in different ways (cell filamentation or cell bloating). We observed that in these conditions lysis plaque enlargement is linked to the host’s morphological changes. We conclude this work with a mathematical model that captures such observations and explains the increase in plaque size observed in the presence of antibiotics.

## Author summary

Phages are viruses that infect and kill bacteria. When phages infect bacteria on a solid surface, they create clear zones called lysis plaques, which are circular areas where the bacteria have been killed. These plaques form as the phages replicate and spread outward from a single point of infection. The consistency of the medium, the characteristics of the phage, and the physiology of the bacterial host influence the speed of plaque enlargement as well as their final size.

In current medical treatments, antibiotics and phages are used together to fight antibiotic resistant bacterial infections. Our research focuses on how antibiotics affect phage activity, which is crucial for improving phage therapy.

In this work, we propose that the enlargement of plaques observed in the presence of sublethal concentrations of certain antibiotics is related to changes in the host bacteria’s shape, which affects how quickly the phages spread and diffuse in a semi-hard environment. To test this idea, we studied the epidemics of two different phages (T5 and T7) during *E. coli* MG1655 infection in the presence of antibiotics that affect (or not) cell morphology in different ways (cell elongation or cell bloating). We conclude this work with a mathematical model that captures such observations and explains the increase in plaque size observed in the presence of antibiotics.

## Introduction

Bacteriophages, viruses that parasitize and kill bacteria, are ubiquitous players in every ecosystem on Earth, participating in fundamental biogeochemical processes and driving bacterial evolution through the many complex interactions they entertain with their hosts [1–3]. Yet phage research under laboratory settings is fundamental to gain insight on such dynamics as well as for the development of phage therapy to fight against bacterial pathogens.

When culturing bacteria in solid media, one of the most distinctive manifestations of phage infection is the appearance of a lysis plaque, the circular clearing on a bacterial lawn following phage predation. In fact, the term bacteriophage (literally “bacteria eater”) was coined after the observation of this dramatic phenomenon more than a century ago [4]. During plaque formation, successive cycles of phage replication take place, usually starting from a single infection spot, and propagating radially on a matrix of immobilized bacterial hosts [5]. Many factors are considered to affect plaque size such as the composition and viscosity of the propagation matrix and the characteristic of a given phage, but also parameters related to bacterial host physiology [6]. From a therapeutic point of view, maximizing phage propagation can be an advantage, since it allows the spread within larger infected areas and the predation of larger amounts of bacteria in spatially structured environments such as plant or animal tissues. In this context, it has been documented that the presence of certain antibiotics can boost phage predation resulting in larger lysis plaques on a bacterial lawn, a phenomenon named Phage-Antibiotic Synergy (PAS) [7,8]. As a matter of a fact, PAS has been known for a long time and in the past two decades its study underwent extensive revival due to its relevance for phage applications. In therapy, combined treatments including both phages and antibiotics are frequent in the case of compassionate care and led to the publication of promising case studies [9–11]. Under PAS conditions, bacterial physiology is altered by the presence of antibiotics, allowing for a more efficient propagation of phages. When this propagation takes place in a lawn of bacteria spread out on a solid medium, synergy can be observed as an increase in plaque size, which could be linked to an increased rate of lysis plaque formation. In past publications, synergy was mainly attributed to an increase in phage fecundity, a parameter represented by phage burst-size, which is the number of virions produced per cell [7,12]. However, since plaque formation is strongly influenced by host growth dynamics, a comprehensive model that incorporates host growth and phage infection parameters was still needed. This aspect should not be overlooked in the context of PAS where the host metabolism and physiology are deeply affected by the presence of a given antibiotic. In this work, we characterized the effects of various antibiotics and found that antibiotic-induced morphological changes are key to increase the speed of propagation of phage epidemics. In addition to filamentation, which has been correlated with PAS in several studies [7,12–14], we assessed the role of mecillinam-mediated cell bloating in increasing lysis plaque size. Furthermore, by emulating these two altered morphologies via inhibition of key regulators of bacterial shape by CRISPR interference, we reproduced the increase in lysis plaque size observed during synergy between phages and antibiotics. Our experimental data therefore suggest that the underlying mechanism of PAS is highly dependent on the type of morphological alteration induced by the drugs. We conclude this work with a mathematical model that captures such observations and explains the increase in plaque size observed in the presence of antibiotics. To the best of our knowledge, this is the first experimental work to characterize epidemic dynamics in the context of PAS, taking into account morphological changes associated with antibiotics.

## Results

### Plaque size increases in the presence of sublethal doses of antibiotics that modify cell shape

To approach PAS in a systematic way, we tested if several antibiotics belonging to different families could increase the radii of lysis plaques of phages T5 and T7 infecting *E. coli* MG1655. The antibiotics selected in this study cause either bacterial filamentation by impairing division (ciprofloxacin and ceftazidime), cell bloating (mecillinam), or no effect on the cell morphology (kanamycin and chloramphenicol). In order to measure the influence of cell shape modification on the size of plaques generated by phages, we measured the radii of plaques formed by phages T5 and T7 in the presence of sublethal doses of the antibiotics mentioned above.

**Fig 1:**
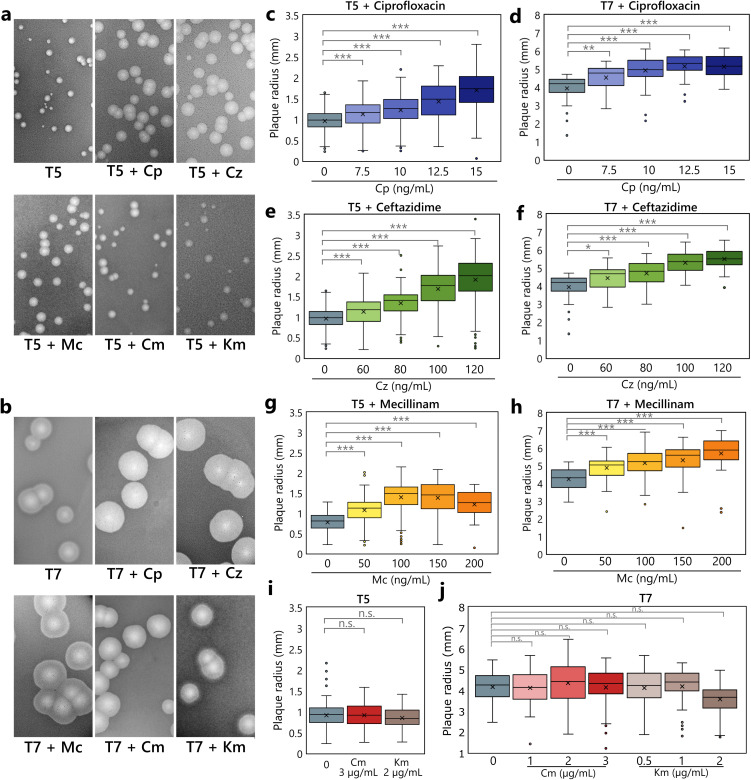
Lysis plaques produced by phages T5 and T7 on *E. coli* MG1655 are enlarged in the presence of sublethal doses of antibiotics. **(a, b)** Aspect of the lysis plaques of phages T5 and T7 at the maximum sub-inhibitory concentration of the tested antibiotics. (c, d, e, f, g, h, I, **j)**. Boxplots of lysis plaque radius measurements at increasing concentrations of: ciprofloxacin (Cp), ceftazidime (Cz), mecillinam (Mc), chloramphenicol (Cm) and kanamycin (Km) for phages T5 and T7. The gradient of concentration used was right below the inhibitory concentration. N = for each condition, between 18 and 414 plaques were measured. Whiskers represent the range of the data within 1.5 times the interquartile range from the lower quartile and upper quartile, mean values are shown as crosses. The asterisks represent significant pairwise comparisons, with significance determined by a linear model (ANOVA followed by post-hoc pairwise comparisons). Three asterisks represent a p-value of less than 0.001, one asterisk represents a p-value of less than 0.05; whereas “n.s.” represents p-values higher than 0.05 in the same test. P-values are listed in S1 Table.

Fig 1a and 1b show the effect of sublethal antibiotic concentrations on the aspect of lysis plaques of phages T5 and T7, respectively. These plaques were obtained through a standard agar overlay assay in the presence or absence of drugs. For each antibiotic, the highest concentration tested corresponds to its maximum sublethal dose (see Material & Methods) which does not lead to a significant reduction in the density or homogeneity of the bacterial lawn in the top agar. For phages T5 and T7 the only antibiotics that induced a significant increase in plaque size were ciprofloxacin, ceftazidime, and mecillinam, whereas neither chloramphenicol nor kanamycin showed such effect.

In order to measure this phenomenon and to determine if the synergistic behavior between phages and antibiotics was dose-dependent, we carried out similar agar overlay assays across a gradient of increasing antibiotic concentrations, measuring the radii of the resulting plaques. The mean radii of lysis plaques produced by phages T5 and T7 increased in the presence of the two filamentation-inducing antibiotics, namely ciprofloxacin and ceftazidime in a dose-dependent manner, compared to the untreated condition (Fig 1). Phage T5, which has a mean plaque radius of 0.88 ± 0.26 mm after 24 hours of incubation in the absence of antibiotics, reached a mean radius of 1.70 ± 0.44 mm (93% increase) and 1.91 ± 0.57 mm (117% increase) in the presence of 15 ng/mL ciprofloxacin and 120 ng/mL ceftazidime, respectively. Similarly, T7 lysis plaques with a mean radius of 4.12 ± 0.69 mm after 24 hours of incubation, showed a maximum radius of 5.13 ± 0.69 mm (25% increase) and 5.49 ± 0.62 mm (33% increase) with 15 ng/mL ciprofloxacin and 120 ng/mL ceftazidime, respectively. Furthermore, a gradual increase in the concentration of mecillinam, which induces cell bloating, also correlated with an increase in the average plaque radius of both phages (Fig 1g and 1h), with a maximum radius of 1.38 ± 0.41 mm (57% increase) and 5.69 ± 0.98 mm (38% increase), for phages T5 and T7 respectively. In this case however, the trend observed with phage T5 indicates that synergy starts to decrease past 150 ng/mL of mecillinam (Fig 1g). Hence, we conclude to a dose-dependent synergistic effect of these antibiotics with two phage species belonging to two taxonomically distant phage families (*Demerecviridae* for T5 and *Autotranscriptaviridae* for T7). In contrast, no significant increase in plaque size was observed at various sublethal concentration of chloramphenicol and kanamycin (Fig 1i and 1j), evidencing the absence of effect on phage propagation in both cases. We also noticed that at the highest concentrations we used, these two antibiotics begin to impair growth without significantly modifying plaque sizes (Fig 1i, 1j, and S1 Fig). In the presence of kanamycin 2 µg/mL we even start to observe an antagonistic effect of this translation inhibitor (Fig 1j). Such antagonistic interactions between phages and some antibiotics have already been documented [15,16]. Altogether, these results highlight a strong correlation between the increase in propagation speed of phages T5 and T7 and the presence of certain types of antibiotics, notably ciprofloxacin, a DNA gyrase inhibitor and known inducer of cell filamentation through the activation of the SOS response, cephalexin and mecillinam, which both belong to the β-lactams family.

### Two morphological changes, filamentation and cell bloating, correlates with an increase in plaque size

**Fig 2:**
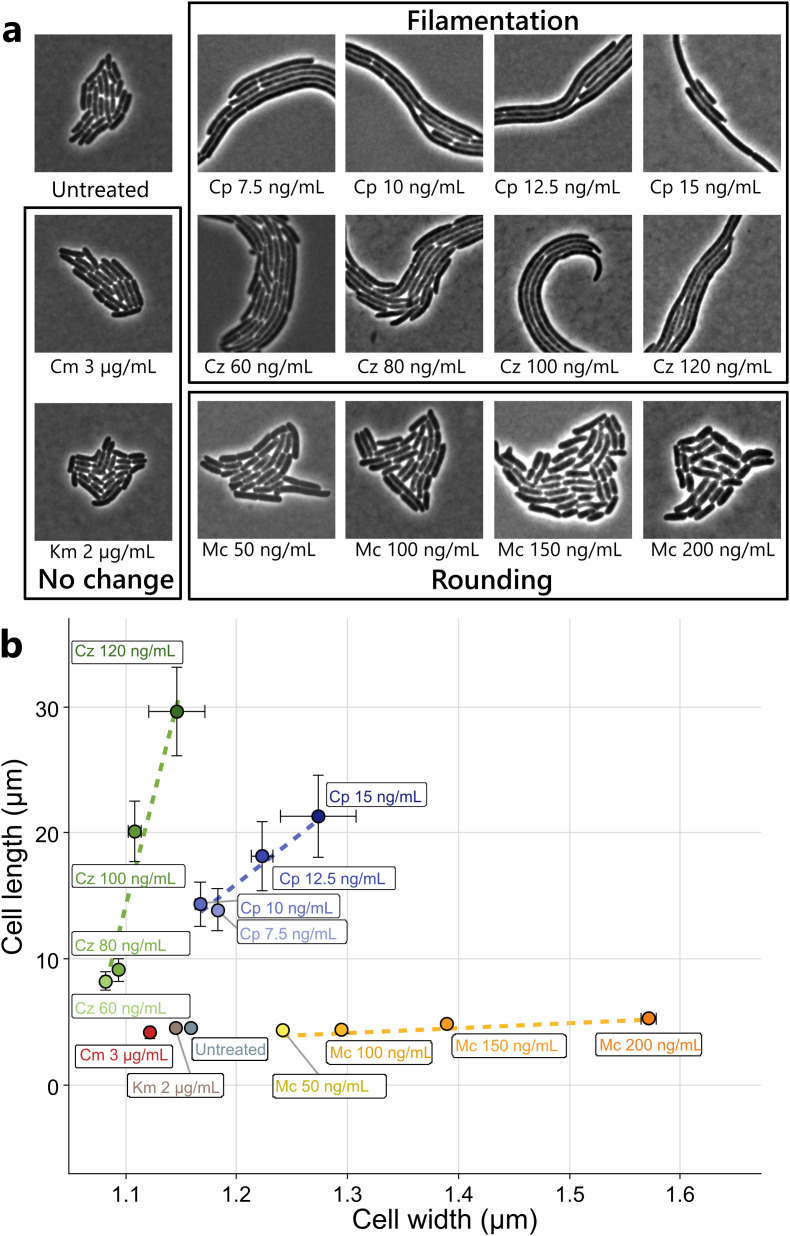
Morphological effects of sublethal antibiotics in E. coli MG1655. **(a)** Phase-contrast microscopy of *E. coli* microcolonies trapped in a bidimensional LB-agarose 1% pad, in the presence of the same antibiotic concentrations tested in Fig 1. **(b)** Scatter plot showing the mean bacterial cell width and length of E. coli populations treated with increasing concentrations of ciprofloxacin (Cp), ceftazidime (Cz), mecillinam (Mc), chloramphenicol (Cm) and kanamycin (Km). N = for each condition, between 171 and 2684 individual bacteria were measured. Whiskers represent the standard error of the mean.

To evaluate the extent of morphological changes in *E. coli* cells triggered by the presence of the synergistic drugs in our experimental conditions, we measured morphological changes of individual bacteria in cultures treated with increasing sublethal antibiotic concentrations. Fig 2a showcases phase contrast microscopy images of bacterial microcolonies exposed to the previously described antibiotics. Morphological changes such as cell filamentation (ciprofloxacin and ceftazidime) or cell bloating (mecillinam) were observed and increased in a dose-dependent manner. As expected, control antibiotics (chloramphenicol and kanamycin) did not provoke any morphological modifications. To quantify these observations, large populations of bacteria were imaged and measured under the same drug concentrations. Fig 2b scatterplot summarizes such effects through two morphological descriptors: mean cell width and mean cell length. As previously observed, the antibiotic ceftazidime increased cell length in a dose-dependent manner, without significantly modifying cell width. The increment on average length observed at the highest concentration of the drug was of 550% compared to the untreated condition. Ciprofloxacin also increased cell length in a dose-dependent manner with a maximum increase of 300%. Additionally, ciprofloxacin moderately impacted width, with a maximum increase of 10%. On the other hand, mecillinam caused an increase in cell width progressively with increasing doses. At the highest sublethal dose (200 ng/mL), we observed a maximum increase in mean cell width of 36%, without affecting significantly cell length (Fig 2b). Finally, neither chloramphenicol nor kanamycin significantly affected mean cell length or width, even at the highest sublethal concentrations (3 and 2 µg/mL, respectively). To reconnect these trends to the morphological changes induced by antibiotics, we then plotted plaque radius according to cell length or cell width (S2 Fig), and in all cases we observed a clear trend linking plaque size and cell length or width. Taken together, our results showed that the degree of morphological change correlated with the strength of synergy measured by plaque size radii.

### Changes in morphology correlate with an increase in the size of lysis plaques on *E. coli*

**Fig 3:**
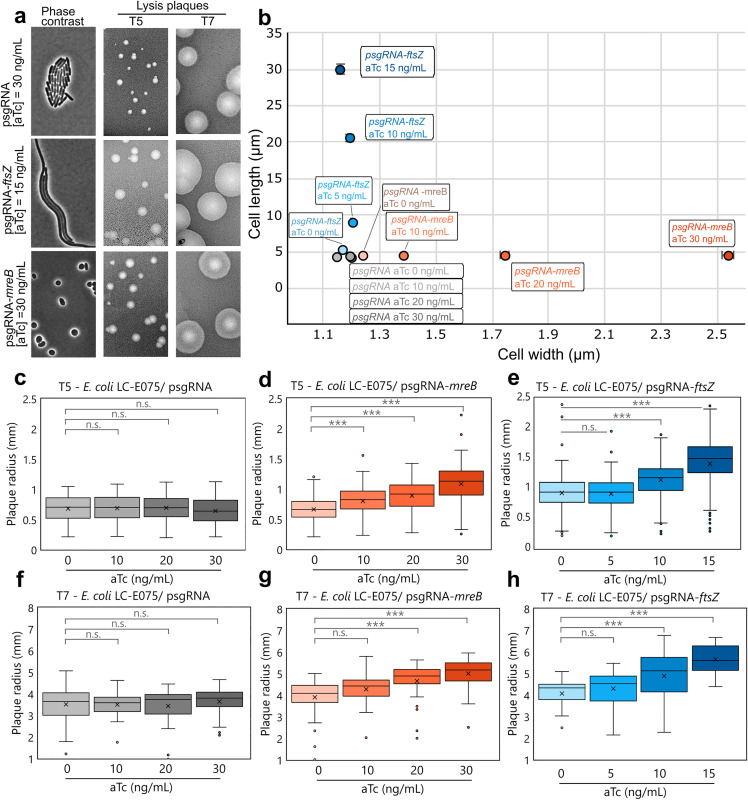
dCas9/sgRNA-driven morphological changes in bacteria produce larger lysis plaques. (a) Effect of dCas9/sgRNA repression of key regulators of bacterial morphology and its impact on plaque size of phages T5 and T7. First column: phase-contrast microscopy of *E. coli* LC-E75 microcolonies under an agarose pad. Each image showcases a microcolony of the LC-E75 strain carrying a sgRNA that either does not target any *E. coli* locus (psgRNA), targets *ftsZ* open reading frame (psgRNA-*ftsZ*) or target *mreB* open reading frame (psgRNA-*mreB*), in the presence of aTc at maximum sublethal dose. Second and third columns, images of lysis plaques of phages T5 and T7 on LCE75 carrying the same sgRNAs and under the same induction concentrations. (b) Scatter plot showing the mean bacterial width and length of *E. coli* LC-E75 in the presence of dCas9 and the sgRNAs mentioned above, at increasing inducer (aTc) concentration. N = For each condition, between 369 and 4,286 individual bacteria were measured, a total of 26,978 cells analyzed. Whiskers represent the standard error of the mean. (c, d, e) Measurements of phage T5 lysis plaque radii on *E. coli* LC-E75 with either a non-targeting sgRNA, sgRNA-*mreB*, or sgRNA-*fstZ*. (f, g, h) Measurements of phage T7 on *E. coli* LC-E75 with either a non-targeting sgRNA, sgRNA-*mreB*, or sgRNA-*fstZ*. N = For each condition, between 21 and 352 plaques were measured. Whiskers represent the range of the data within 1.5 times the interquartile range from the lower quartile and upper quartile, mean values are shown as crosses. The asterisks represent significant pairwise comparisons, with significance determined by a linear model (ANOVA followed by post-hoc pairwise comparisons). Three asterisks represent a p-value of less than 0.001, one asterisk represents a p-value of less than 0.05; whereas “n.s.” represents p-values higher than 0.05 in the same test. P-values are listed in S1 Table.

Our results suggest that two completely different morphological changes, filamentation and cell rounding, correlate with an increase in the mean radius of lysis plaques. To demonstrate whether filamentation or cell bloating alone were sufficient to induce an enlargement of phage lysis plaques, we aimed at emulating these morphological perturbations in *E. coli* without using the antibiotics mentioned earlier. To achieve this, we resorted to a system based on the “dead” Cas9 (dCas9) enzyme, a variant of the well-known Cas9 from *S. pyogenes* inactivated in its endonuclease activity [17]. This variant is still able to use a sgRNA to interrogate and bind to the target DNA. However, due to its abolished catalytic activity, the dCas9-sgRNA complex remains bound to the target dsDNA molecule without cleaving it [18]. If the sgRNA is designed to target a particular promoter or the coding sequence of the downstream ORF, the binding of the dCas9 will physically block the gene’s transcription by preventing RNA polymerase binding or elongation. The dCas9 encoding gene is carried by the *E. coli* strain LC-E75, which is an *E. coli* MG1655 derivative encoding the *dcas9* gene under the control of a promoter inducible by anhydrotetracycline (aTc), whereas the sgRNA is constitutively produced from the plasmid psgRNA. The specificity of the targeted gene was conferred by the 20 complementary nucleotides cloned within the sgRNA on the plasmid (see Materials and Methods). We inferred that by impairing the expression of key genes involved in cell morphology we could mimic cell elongation or cell rounding triggered by the above-mentioned antibiotics. FtsZ is a major component of the division machinery in many bacterial species and its depletion blocks cell septation and produces long filaments [19], emulating ceftazidime- and ciprofloxacin-induced morphological changes. Likewise, we expected that impairing *mreB* gene expression, that encodes a key component of the bacterial elongasome and leads to round and bloated cells [20], would mimic mecillinam-induced morphological changes. Fig 3a left column shows cell morphology after two hours induction by aTc in the case of a non-targeting sgRNA (Fig 3a, upper panel), a *ftsZ*-targeting sgRNA (Fig 3a, center panel) and a *mreB*-targeting sgRNA (Fig 3a, lower panel). As expected, filamentation and cell bloating were induced when *ftsZ* or *mreB* expression was impaired, respectively. The negative control showed that the expression of *dCas9* alone did not modify cell morphology. These results demonstrated that we could selectively mimic antibiotic-induced cell morphology changes. We thus proceeded to the measurement of lysis plaques with both T5 and T7 as previously described (Fig 3a, middle and right columns, respectively). With both T5 and T7 we observed that the two dCas9/sgRNA mediated morphological changes correlated with a strong increase in the size of lysis plaques compared with the condition with non-targeting sgRNA, reminiscent of increase observed with ceftazidime, ciprofloxacin and mecillinam. As above, we also included in S2 Fig the plots linking plaque size and cell morphology. These results thus support the hypothesis that either filamentation or bloating by themselves allowed increased phage predation.

Since we previously observed that cell morphology as well as lysis plaques size vary with increasing concentrations of ceftazidime (cell elongation) and mecillinam (cell bloating) (Fig 2b), we quantified the effect of *ftsZ* and *mreB* gene repression using the same morphological descriptor (*i.e.*, cell width, cell length and lysis plaque radius) 24 hours post-infection in the presence of increasing concentrations of inducer. Fig 3b shows the average bacterial width and length measured in strains expressing dCas9 with a sgRNA targeting either *ftsZ*, *mreB*, or a control sgRNA that did not target any *E. coli* gene. In the presence of a spacer targeting *mreB* (cell bloating), we observed an aTc dose-dependent increase in cell width reaching a maximum of 2.54 ± 0.44 µm at 30 ng/mL aTc compared to 1.20 ± 0.07 µm in the strain carrying the empty vector at the same induction level (112% increase). We measured a strong increment in cell length in the presence of an *ftsZ*-targeting spacer (cell filamentation), which is also proportional to the aTc inducer concentration increased. Fig 3b shows an average length for *ftsZ*-repressed cells of 5.31 µm, 8.95 µm, 20.60 µm, and 29.93 µm for *dCas9* induction with aTc = 0 ng/mL, 5 ng/mL, 10 ng/mL, and 15 ng/mL, respectively. The maximum length reached almost 30 µm when aTc was added at the maximum concentration (15 ng/mL) compared to 4.35 ± 1.32 µm in the control experiment with the non-targeting sgRNA (996% increase).

dCas9-induced morphological changes properly imitate the effects of the synergistic antibiotics we tested previously. Firstly, the increase in length obtained with dCas9-mediated repression of *ftsZ* is comparable to the one observed in the presence of ceftazidime and 1.4 more important than in the presence of ciprofloxacin. Secondly, the increase in bacterial width as a consequence of *mreB* inhibition is 1.6 times the one observed with mecillinam. We cannot exclude at this point that the presence of antibiotics might disturb other key cellular processes leading to cell death before achieving the major changes in cell morphology observed upon inhibition of *ftsZ* or *mreB* genes.

We thus successfully adapted a dCas9-based tool to reproduce two morphological effects, filamentation and bloating, observed upon sublethal antibiotic treatments. We then wondered if such morphological changes, obtained in the absence of antibiotics, could account for enlarged lysis plaques previously observed in the presence of antibiotics (Fig 1). To evaluate this, we carried out the standard agar overlay assay as described before with increasing concentrations of the inducer to evaluate the size of the lysis plaques during infection by phages T5 and T7 (Fig 3c-3h). When a non-targeting psgRNA was used, we did not observe any significant increase of the lysis plaque radii either with T5 or T7, whatever the inducer concentrations (Fig 3c and 3f). In the presence of *mreB*-targeting or *ftsZ*-targeting psgRNA, we observed an aTc dose-dependent increase in mean lysis plaque radii for T5 (Fig 3d and 3e) and T7 (Fig 3g and 3h). When cell bloating is induced by repressing *mreB* expression, lysis plaque radii increase by 63% and 28% compared to the control experiment for T5 and T7, respectively (Fig 3d and 3g). When cell filamentation is induced by repressing *ftsZ* expression, lysis plaque radii increase by 53% and 38% compared to the control experiment for T5 and T7, respectively (Fig 3e and 3h). These results suggest that antibiotic-mediated synergies reported in Fig 1 are chiefly driven by bacterial morphology changes (filamentation for ceftazidime and ciprofloxacin, bloating for mecillinam) and not by other effects of the antibiotic on the host. In other words, our findings suggest that cell bloating or filamentation, induced either by antibiotics or by the use of the dCas9/sgRNA system, would be the main factor responsible for the increase in both T5 and T7 lysis plaques size.

### Filamentation increases phage burst size through different mechanisms

In the previous sections, we used a dCas9/sgRNA system to block the transcription of genes involved in the maintenance of *E. coli* shape in order to demonstrate that both bacterial filamentation and bacterial bloating were sufficient to increase the plaque size of phages T5 and T7. After having established a link between morphological changes and increased plaque size, we questioned the underlying mechanisms of plaque enlargement driven by two contrasting morphological effects. To test this, we should determine whether filamentation and bloating impact phage predation in a similar way or contribute to plaque enlargement through distinct mechanisms.

**Fig 4:**
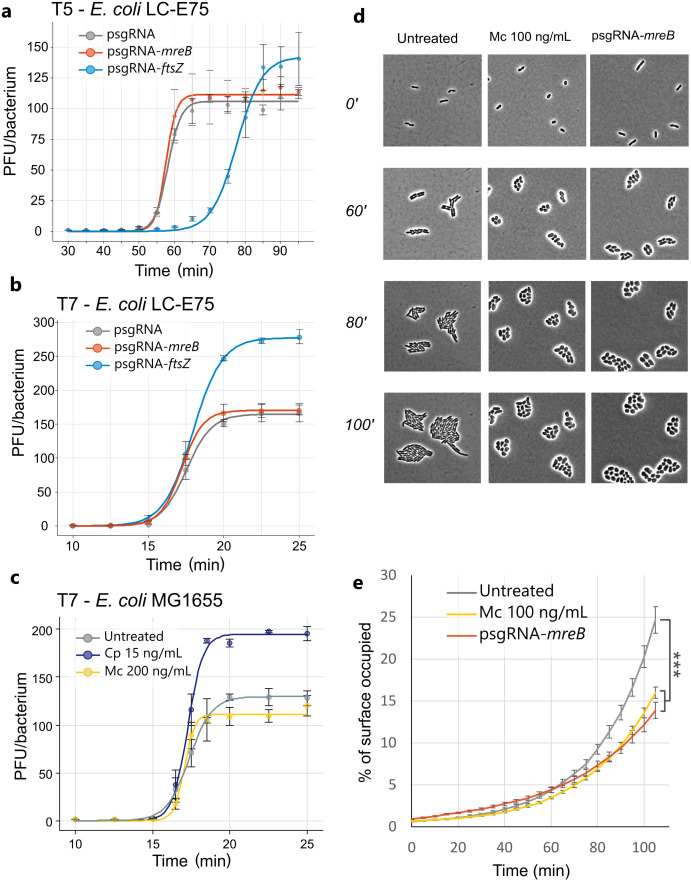
Effects of dCas9-mediated morphological changes on key parameters influencing epidemic spread. **(a, b)** One step growth curve of phage T5, and phage T7 in *E. coli* LC-E75 displaying different morphological changes. Time = 0 corresponds to the time of phage-bacteria mixing. Induction of the morphological change by dCas9 transcription inhibition was carried out for 2 hours prior to the beginning of the infection. Morphological changes at the time of the phage addition were confirmed by phase-contrast microscopy. Concentrations of the inducer were the maximum sublethal concentrations tolerated by the strain. N = 3 curves per condition. Error bars represent the standard error of the mean. **(c)** One step growth curve of phage T7 in *E. coli* MG1655 in the presence of the antibiotics mecillinam and ciprofloxacin at their maximum subinhibitory concentrations. Time = 0 corresponds to the time of phage-bacteria mixing. **(d)** Phase contrast microscopy images of time lapse growth of bacteria in the presence of mecillinam in *E. coli* MG1655 or after repression of the *mreB* gene by the dCas9/sgRNA in strain LCE-75. Cells were cultured in liquid medium and pre-treated with the antibiotic or the inducer one-hour prior sampling and microscopy imaging. **(e)** Percentage of a given surface area (900 µm²) occupied by microcolonies of *E. coli* under different conditions over 105 minutes of growth at 37°C. N = between 30 to 43 microcolonies were followed and measured per condition. Error bars represent the standard error of the mean. Three asterisks represent a p-value of less than 0.001 for an upper-tailed t-test comparing values at time = 105 minutes.

In order to assess the impact of a given morphological change in the replicative cycle of phages T5 and T7, we carried out one-step growth curve experiments in the presence of dCas9-mediated morphological changes achieved through inhibition of *ftsZ* or *mreB* gene transcription, and in the presence of either ciprofloxacin or mecillinam. Fig 4 showcases replication of phages T5 and T7 at 37°C in liquid LB medium. For phage T5 (Fig 4a) a latent period of 50 minutes and a burst size of 110 PFU/bacterium were observed in the presence of a non-targeting sgRNA, in agreement with the literature [21]. When filamentation was induced through the inhibition of *ftsZ* transcription, a delay of about 15 min in the latent period was observed as well as an increase of 30% in burst size. The cause of the increase in progeny size might be attributed to a delay in the period of intracellular phage assembly, which allowed a larger pool of phages to be produced and maturated per cycle. In contrast, cell bloating achieved through *mreB* inhibition neither modified the latent period nor the burst size of phage T5. This suggests that plaque enlargement under bloating is not a consequence of increased phage fecundity.

We also compared replication of phage T7 under the same conditions (Fig 4b), and in the presence of ciprofloxacin or mecillinam (Fig 4c). In the absence of morphological alteration, phage T7 displayed a latent period of 15 minutes and a burst size of approximately 120–160 PFU/bacterium, which corresponds to expected burst sizes [21,22]. Upon *ftsZ*-mediated filamentation, burst size increased dramatically compared to the control with a non-targeting spacer. Very similar results were obtained in the presence of ciprofloxacin (Fig 4c). However, unlike phage T5, filamentation did not impact T7’s latent period. The production of a larger phage progeny within a similar latent period could indicate that filamentous bacterial cytoplasm may allow for larger phage factories to be formed prior to lysis, as observed previously with another phage model HK620 [13]. Finally, as observed for phage T5, bacterial bloating caused by either *mreB* inhibition or mecillinam addition, neither affected significantly the latent period nor the burst size of phage T7, suggesting a different mechanism accounting for enhanced phage predation. It should be mentioned as well that these experiments are exclusively performed in liquid and agitated medium.

### Cell bloating may improve phage diffusion by reducing phage-bacteria interactions

Since the replicative cycles of phages T5 and T7 were not modified when bacteria suffered cell bloating, we hypothesized that the increase in plaque size was rather due to changes in the dynamics of phage-bacteria interactions during propagation in a semi-solid matrix [23]. These changes could improve phage diffusion in the bacterial lawn matrix. To test this hypothesis, we measured the packing of *E. coli* microcolonies either in the presence of mecillinam or upon *mreB* expression inhibition (Fig 4c and 4d). As observed in Fig 4c, whatever the cause of cell bloating, microcolonies tend to adopt round and compact arrangements over time compared to the untreated condition. To quantify this, we measured the percentage of bidimensional space occupied by each microcolony over time (Fig 4d): microcolonies composed of bloated bacteria tend to occupy around 38% less space compared to microcolonies composed of regular-shaped cells after 100 minutes of incubation. The boost of phage propagation could then be explained as follows: a reduction of the fraction of space occupied by the host is predicted to increase the diffusion of viral particles [6]. Consequently, in the presence of compact arrangements of bacteria allowed by cell bloating, phages can diffuse and penetrate further in the bacterial matrix before adsorbing to a non-infected host, increasing the overall speed of phage propagation and then the lysis plaque radius.

### Recording phage T7 propagation

In order to study the propagation of phage epidemics we followed the dynamics of phage propagation by recording lysis plaque expansion on plates with time lapse imaging. In the case of T7 this can be conveniently carried out over relatively long periods of time due to the phage ability to infect bacterial cultures that have entered into the stationary phase [24]. We thus monitored T7 lysis plaque formation in the presence of increasing concentrations of mecillinam (inducing cell bloating) and ciprofloxacin (inducing cell filamentation). S3 Fig shows the resulting kinetics of plaque enlargement.

In all tested conditions, these kinetics were biphasic. The first phase lasted about 14 hours and the propagation rate - measured as the slope of plaque formation kinetics - was maximum and fairly constant for all antibiotic concentration tested. This initial phase corresponds to the bacterial lawn exponential growth phase. The shift to the second kinetic regimen follows the entry of the bacterial lawn into the stationary phase. During this second phase the propagation rate decreased compared to the initial phase but in an antibiotic dose-dependent manner for the two tested antibiotics. Compared to the initial phase where propagation rates are slightly affected by the antibiotic concentrations (S3 Fig), we observed during this second phase that rates increased with the antibiotic concentrations. These results are in accordance with increases in plaque size measured at a fixed time post infection we previously observed (Fig 1). Altogether, these kinetics profiles suggest that the increase in plaque size was mostly due to an accelerated T7 propagation taking place in the mature bacterial lawn stimulated by the addition of mecillinam and ciprofloxacin.

### Cell Morphology-driven mathematical model of phage lysis plaque expansion

This study aims to investigate how antibiotic-induced morphological changes in bacteria influence phage predation and lysis plaque expansion. Experimental results suggest that alterations in bacterial shape and size impact the spread of phage infections, prompting the need for a theoretical model that takes into account the effect of such morphological changes on plaque size expansion rates. Thus, we develop a simplified mathematical model to identify key parameters and explore underlying principles that govern the interaction between bacterial morphology and phage propagation.

#### Theoretical framework: Conceptual overview.

The proposed model describes changes in the rate of phage plaque propagation (terminal velocity) as a function of bacterial and phage physiological parameters. At its core, we consider two states of the phage particles: “free” and “adsorbed” phages. Free phages diffuse isotropically on the substrate and encounter bacterial cells and are eventually adsorbed with rates proportional to available average surface area of the bacteria. The adsorbed phages multiply with rates proportional to the length of the bacteria (which emulates multiple focal points adsorbed in long cells). The adsorbed phages are then released during lysis of the bacteria as free phages. Assuming the adsorption and release rates of adsorbed phages are faster than the diffusion of the free phages, we can obtain a dynamical equation of the total concentration of phages as shown in Equation 1 in the supplementary text. Then we seek a traveling wavefront solution for the total phage dynamics to obtain an analytical expression for the minimum speed of the travelling wavefront as a function of average morphological parameters of the bacterial cells, phage physiological parameters and external parameters reflected by the biomass growth rate. For phages with negligible deactivation rate (as in the case of T7 phage), we obtain the following expression for the speed of the travelling wave-front:


$$c^{\text{2}}\geq \text{4}\delta \frac{\gamma l\lambda \sigma V}{(\text{1}+\lambda \sigma V{)}^{\text{2}}}$$ (1)

with $\lambda =\frac{\alpha /\beta }{\text{1}-\gamma l/\beta }$. The above equation gives a lower bound to the speed of travelling lysis wavefront, whenever a travelling wavefront solution exists. The parameters that affect the speed are the diffusion constant of the free phages on the substrate (δ), the adsorption rate (α), an average release rate (β) which is not the burst rate, replication rate of phages per unit cell length (γ), σ is the surface area-to-volume ratio which, in the case of sphero-cylindrical cell shape, it can be equated to the cell width, w, as σ~ 1/w. Cell length is denoted by l and the biomass encountered by the travelling wavefront, V.

Assuming a slow growth of biomass, the terminal speed *c* at the time the biomass reaches capacity ( $V_{max}$ such that $\lambda \sigma V_{max}$>>1) is $c^{\text{2}}\;\geq \;\text{4}\delta \frac{\gamma l}{\left(\lambda \sigma V_{max}\right)}$ ~ $\;\frac{\text{4}\delta \gamma \beta }{\alpha V_{max}}lw(\text{1}-\gamma l/\beta )$. For a given phage the parameters α, β,γ and δ remain a constant. Further, in case of antibiotic-treated cells with similar nutrient and experimental conditions the biomass capacity $V_{max}$ should remain constant as well, even though the cell growth rate may. To account for variability in experimental conditions we keep the $V_{max}$ as a variable for different experiments.

Now, taking the ratio of speeds in an experimental case $c_{T}$ (*T* for antibiotic-treated cells) to the speed of untreated host $c_{u}$ (*u* for untreated cells) we cancel out the constants, leading to the expression:


$${\left(\frac{c_{T}}{c_{u}}\right)}^{\text{2}}=\frac{V_{max,u}}{V_{max,T}}\frac{l_{T}w_{T}}{l_{u}w_{u}}\frac{\text{1}-\frac{\gamma l_{T}}{\beta }}{\text{1}-\frac{\gamma l_{u}}{\beta }}$$ (2)

It can be noted that in case of phages where replication rate is small (γ<< β) this ratio only depends on the product of relative length and width of the cells.

To compare the theoretical model with experiments, we measured the rate of T7 plaque formation in the presence of mecillinam and ciprofloxacin, two antibiotics that accelerate phage propagation at synergistic antibiotic concentrations, and studied the physiological changes induced in phage and bacteria.

#### Comparison with experimental data.

We recorded lysis plaque radius ( $r-r_{\text{0}})$ enlargement kinetics on solid media for 28 h for untreated cells and cells treated with increasing concentrations of mecillinam (0 – 70 ng/mL) inducing cell bloating (S4a Fig) or ciprofloxacin (0 – 8 ng/mL) inducing cell filamentation (S4b Fig). As mentioned earlier, these kinetics are biphasic with a rapid initial plaque expansion followed by a slow down after approximatively 16 h. We defined for each kinetics the terminal velocity c ( $c_{T}$ for treated cells and $c_{u}$ for untreated cells) as the slope of slow phase derived from the experimental kinetics. We then plotted the squared relative terminal velocity ${\left(\frac{c_{T}}{c_{u}}\right)}^{\text{2}}$against the relevant morphological parameter for the two cases.

#### Mecillinam and cell bloating (Increased width).

**Fig 5:**
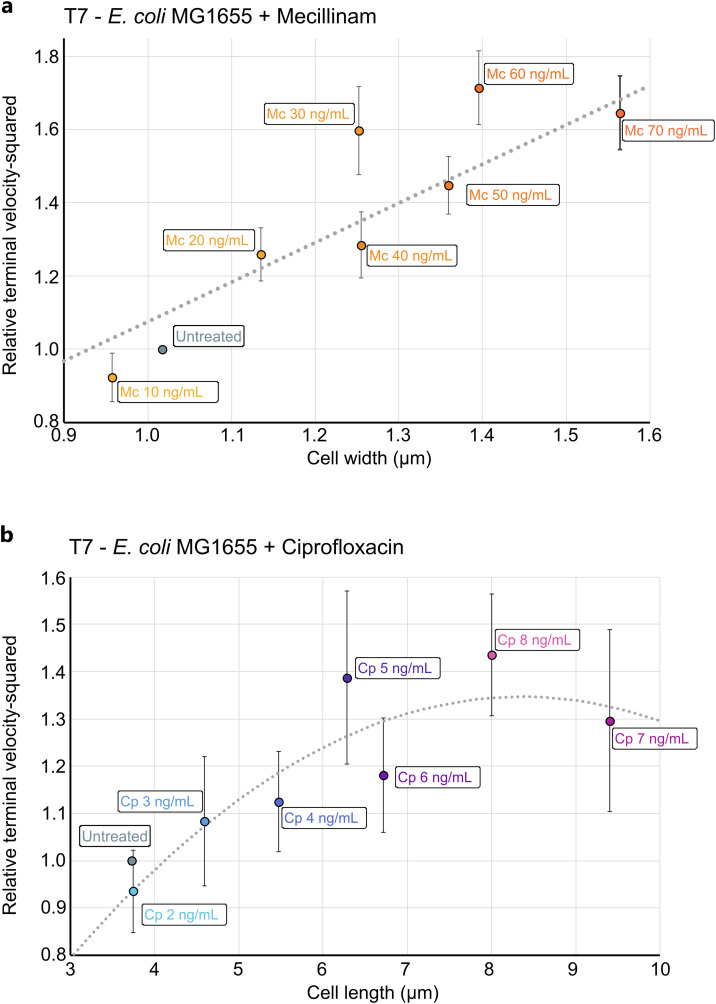
Relative squared speeds of expanding plaques as a function of changes in morphological parameters of cell in mecillinam and ciprofloxacin treated cells. The theoretical model leads to an analytical expression of relative squared speeds as a function of morphological parameters (length and width) and the phage physiological parameters as given in Equation 2. For mecillinam-treated cells, bacterial length remains unchanged while width increases, resulting in a linear relationship between squared speed and width (Eq. 3), as shown in Fig 5a. The dots represent experimentally measured speeds as a function of cell width, which align well with the model’s predictions. Similarly, in ciprofloxacin-treated cells, bacterial width remains constant while length changes (Eq. 4). In this case, the experimentally determined relative squared speed (dots) is plotted against cell length, and the model’s fit is shown in Fig 5b.

When cell length l remains constant as it is the case with mecillinam (Fig 2) or when *mreB* gene expression is repressed (Fig 3), Equation 2 can be simplified by noting that $l_{T}\sim l_{u}$, leading to::


$${\left(\frac{c_{T}}{c_{u}}\right)}^{\text{2}}=\frac{w_{T}}{w_{u}}=a_{\text{1}}*w_{T}$$ (3)

where the constant $a_{\text{1}}\sim \;\text{1}/w_{u}\;\frac{V_{max,u}}{V_{max,T}}$. This constant also takes into account experimental discrepancies that may lead to the different biomass capacity for treated and untreated conditions. The slope (speed) derived from the experimental kinetics are computed and the squared relative terminal velocity ${\left(\frac{c_{T}}{c_{u}}\right)}^{\text{2}}$ is plotted against against cell width w for mecillinam. The linear model in Equation 3 is fit to the experimental data to estimate $a_{\text{1}}=\text{1}.\text{0}\text{8}\pm \text{0}.\text{5}\text{8}\;{\mu m}^{-\text{1}}$. The experimental data and the linear fit are shown in Fig 5a.

#### Ciprofloxacin and cell filamentation (Increased Length).

The cell width remains constant in the case of ciprofloxacin (Fig 2) or when *ftsZ* gene expression is repressed (Fig 3), i.e, $w_{T}\sim \;w_{u}$. Regrouping the terms with constants and lengths of untreated cells into new constants $b_{\text{1}}$and $b_{\text{2}}$, Equation 2 can be written as:


$${\left(\frac{c_{T}}{c_{u}}\right)}^{\text{2}}=b_{\text{1}}*l_{T}*(\text{1}-b_{\text{2}}*l_{T})$$ (4)

with $b_{\text{1}}\sim \frac{\text{1}}{l_{u}(\text{1}-\frac{\gamma }{\beta }l_{u})}\;\frac{V_{max,u}}{V_{max,T}}$ and $b_{\text{2}}=\frac{\gamma }{\beta }$.

According to the above equation, we expect the ratio of squared speeds ${\left(\frac{c_{T}}{c_{u}}\right)}^{\text{2}}$to follow a quadratic trend which increases with cell length l, reaches a maximum at l=β/2γ then decreases while cells keep elongating. Experimental data in Fig 5a does exhibit such a trend. Fitting Equation 4 to the experimentally obtained ratio of squared speeds we obtain $b_{\text{1}}=\text{0}.\text{3}\text{4}\text{9}\pm \text{0}.\text{0}\text{1}\text{8}\;{\mu m}^{-\text{1}}$and $b_{\text{2}}=\frac{\gamma }{\beta }=\text{0}.\text{0}\text{6}\text{5}\pm \text{0}.\text{0}\text{0}\text{7}\;{\mu m}^{-\text{1}}$, with a maximum at $l_{max}=\frac{\text{1}}{\text{2}\;b_{\text{2}}}\;\sim \text{7}.\text{7}\;\mu m$.

#### Insight, significance and limitations.

Despite its simplicity, our theoretical model aligns well with experimental observations in both mecillinam and ciprofloxacin treatments, suggesting that bacterial morphology alone can largely account for variations in PAS efficiency. Specifically, phage lysis plaque expansion kinetics are governed by antibiotic-induced changes in bacterial shape. The model successfully captures the trends observed: PAS efficiency continues to rise with increasing cell width (as seen in mecillinam-treated cells, Fig 5a) but reaches a maximum with increasing cell length (as observed with ciprofloxacin, Fig 5b). With the model we infer that in case of filamentous cells a maximum PAS efficiency is reached when cells are of length l=β/2γ ~7.7 μm. This agreement across two distinct cases highlights the predictive nature of this simplified approach, suggesting it may also be used to estimate outcomes in intermediate cases where both length and width changes contribute simultaneously and where the physiological parameters of phages can be estimated.

The simplified model remains analytically tractable because of the following assumptions. Firstly, we assume that adsorption and release are fast relative to phage diffusion. This assumption simplifies the dynamical equation of the total phage concentration. This quasi-steady state approximation is valid when adsorption and release act on timescales shorter than the diffusion timescale within a relevant length scale *L*. For example, taking typical free diffusion rates between 2–400 µm^2^/min [5,25] and a latent period of 30 min as timescale for adsorption and release, the diffusion time scale *L*^2^/δ should be larger, leading to a length of ~ 10–100 µm above which this assumption remains valid. Thus, it may fail in conditions where adsorption-release rates are slower or when host cells are sparse, where free phages diffuse significant distances before interacting with cells. Secondly, the assumption that biomass volume encountered by the lysis wavefront changes much slower than the wavefront speed only remains valid when the biomass growth rate is much lower compared to the speed of the travelling wavefront. Furthermore, the latent period is not explicitly included. However, the parameters representing the average replication rate per unit length (γ) and the average release rate (β) may indirectly account for this effect. A large β and small γ can phenomenologically represent a long latent period.

By structuring the model around morphology of cells (length and width), physiological parameters of phages (adsorption, replication and release rates) and external dependent parameters like biomass growth rate, we aim to provide a clear, intuitive understanding of the link between bacterial morphology and phage propagation. The derivation and effect of the change in parameters on the speed of travelling wavefront are presented in S4 Fig.

## Discussion

The objective of the present study was to characterize the synergy taking place between two taxonomically distant bacteriophage species (T5 and T7) and several antibiotics with contrasted effects on bacterial morphology. The rationale was to determine whether synergy can take place through a common mechanism for different antibiotic classes, or on the contrary through distinct effects in the phage-bacteria system. To achieve this, we used different classes of antibiotics, namely β-lactam (ceftazidime, a cephalosporin and mecillinam, an aminopenicillin), fluoroquinolone (ciprofloxacin), aminoglycoside (kanamycin) and chloramphenicol. We measured phage-antibiotic synergy (PAS) as the increase of the size of T5 and T7 lysis plaques on lawns of *E. coli* MG1655 undergoing antibiotic stress. The range of antibiotic concentrations we used was right below the threshold of growth inhibition of the bacterial lawn. Under these conditions, we first observed that the tested antibiotics could be classified into two families regarding PAS (Fig 1). The first one (ceftazidime, ciprofloxacin and mecillinam) regroups antibiotics eliciting a dose-dependent enlargement of phage lysis plaques compared to the untreated condition. The second one (kanamycin and chloramphenicol) regroups antibiotics that do not modify the size of the phage lysis plaques. Results were consistent for both bacteriophages T5 and T7 although they belong to taxonomically distant families (*Demerecviridae* for T5 and *Autotranscriptaviridae* for T7) as well as for phage HK620 (belonging to the *Lederbergvirus* genus) in a previous work [13]. We thus successfully set up a standardized method to screen and quantify the PAS effect for various combinations of phage/ antibiotic.

Since we previously evidenced that cell filamentation is a key driver of successful phage predation in the PAS context we examined the consequences of morphological alteration of bacterial cells on phage predation in a broader context [13]. Indeed, the antibiotics we tested can now be classified into three families (Fig 2). The first one comprising ciprofloxacin and ceftazidime induces *E. coli* cell filamentation. The second one comprising mecillinam induces cell rounding. Both phenomena take place in a dose-dependent manner and in the exact same range of concentrations where phage plaque enlargement occurs (Fig 3). The third family including kanamycin and chloramphenicol does not elicit any cell morphological change. One has to note that the three synergistic antibiotics (ceftazidime, ciprofloxacin and mecillinam) belong to two different classes (β-lactam and fluoroquinolone) and that two molecules belonging to the same class (β-lactam) triggers opposite morphological changes (cell filamentation for ceftazidime and cell bloating for mecillinam). Since in the absence of antibiotic-induced morphological changes no synergistic effect is observed, we hypothesize a direct correlation between morphological alteration and accelerated phage propagation rather than with the direct molecular mechanisms specific to each antibiotic.

In order to prove that filamentation and cell rounding were necessary and sufficient to enhance phage propagation, we used a dead-Cas9 system to trigger such altered morphologies. By downregulating the expression of key genes responsible for the maintenance of *E. coli* cell shape (*ftsZ* for cell filamentation and *mreB* for cell rounding), we successfully emulated cell filamentation and cell rounding without the requirement of antibiotics. Strikingly, the effects of cell morphology alteration on phage predation were reminiscent of those observed in the presence of the antibiotics and proportional to the extent of the morphological changes. Through this, we confirmed that synergy happens when bacterial shape is modified into filaments or spheres.

The link between bacterial morphological changes in PAS, as measured from top agar assays, has been emphasized in several studies since its re-discovery [7,12]. Remarkably, synergistic interactions were observed in the bacterial lawn regions immediately adjacent to the antibiotic inhibitory halos, after which plaque size returned to normal. The existence of an appropriate range of antibiotic concentrations where plaque enlargement takes place suggests the role of major physiological effects in the host that take place only at high but still sublethal drug concentrations. In the present study, the choice of liminal subinhibitory concentrations allowed us to reproduce such an effect in a controlled way. Comeau et al. studied synergy between filamentation-inducing antibiotics, and a phage (φMFP) infecting a uropathogenic *E. coli* strain [7]. They suggested that the mechanistic basis for plaque enlargement was the net increase in phage progeny experienced upon antibiotic-induced filamentation. As a result, they proposed that an enlarged bacterial cytoplasm could provide more biosynthetic material and allowed the production of larger phage progenies. In a more recent work, an alternative explanation was suggested, based on the observation that filamentation delays the lysis of phage T4, with a consequent increase in burst size [12]. Although both studies are based on taxonomically distantly related phages, they both agree on the correlation between cell filamentation and PAS on the one hand and on the increase in the burst size to explain lysis plaque enlargement. Through careful investigation of T5 and T7 viral cycles with dCas9-induced elongated cells (Fig 4) we concluded that the impact of filamentation on phage replication ultimately leads to an increased burst size with both phages. Nevertheless, we observed phage-specific differences. In the case of phage T7, a clear increase of the burst size in the presence of dCas9/*ftsZ*-sgRNA-mediated filamentation was observed, without changing the latent period. As mentioned before, this behavior was observed with phage φMFP infecting *E. coli* in the presence of cefotaxime [7], and more recently with coliphage HK620 in the presence of cephalexin and ciprofloxacin [13]. Conversely, upon bacterial filamentation, T5 displayed an increased burst size as a consequence of a delay in the latent period, which in turns aligns with the “delayed lysis” hypothesis proposed by Kim et al. According to this hypothesis, the concentration threshold required for the holin aggregation and disruption of the bacterial envelope increases as a consequence of membrane enlargement upon filamentation [12]. T4 holin T belongs to the type III class of holins consisting in a single transmembrane domain with two soluble domains that protrude into the cytoplasm and periplasm and play a role in lysis-inhibition [26]. Curiously enough, class III holins are only found in T4-like and T5-like phage families [27], which could explain the similar lysis-delay response upon bacterial filamentation.

In contrast, the drastic change in burst size observed in the presence of filamentation was not observed upon cell rounding (Fig 4). In fact, no significant changes in the lytic cycle of phages T5 and T7 were detected under cell rounding mediated by dCas9/*mreB*-sgRNA expression. Since the timing and productivity of an individual viral cycle is not affected during the epidemic spread whether the cell retains its normal shape or becomes rounded, it is highly likely that the synergistic behavior arose from a change in the dynamics of virion diffusion from one host to the next one. We have evidenced here that in the presence of cell rounding, whether it is caused by the addition of the antibiotic mecillinam or the repression of *mreB*, the microcolonies formed by *E. coli* tend to occupy less space due to a more compact spatial arrangement (Fig 4). As a consequence, this abnormal aggregation pattern significantly reduces the fraction of space occupied by the bacterial populations and increases the free, unoccupied space between microcolonies in the matrix. Ultimately, an increase in the free space between host clusters during phage spread can allow virions to freely diffuse farther away from the epidemic center between each cycle, allowing them to travel longer distances before the encounter of a new non-infected bacterium. These results are in accordance with previous theoretical and experimental work suggesting that a reduction in phage-bacteria interaction could increase phage propagation speed [6,28,29]. Thus, by reducing the likelihood of a phage encounter with a cluster of hosts, viral diffusion is the main parameter accounting for the increase in the propagation speed.

Lysis plaque expansion has traditionally been studied as a diffusion-reaction wave of phages over a bacterial lawn [29,30]. Early heuristic approaches for modelling plaque expansion rates were largely phenomenological [31]. A more mechanistic approach was introduced by Yin & McCaskill [29] who modeled diffusing phages binding irreversibly to susceptible, immobile, and non-dividing bacteria, resulting in the production of new virions. However, this model overlooked bacterial growth and the phage latent period. Later, Fort and Mendez [32] extended this framework by incorporating time delays to account for latent periods, significantly improving predictions of plaque expansion speeds. Building on the reaction-diffusion based models, our study proposes a simplistic model that explicitly incorporates bacterial morphological parameters, cell length and width, which were experimentally measured under different antibiotic treatments. Specifically, our model aims at providing an analytical expression of the speed of the travelling lysis wavefront as a function of morphological parameters of the cells and physiological properties of phage to be able to estimate the speed of the lysis front for different phages and antibiotic treatments.

According to our framework, plaque propagation speed is a function of cell shape (length and width), phage physiological parameters (adsorption, replication, release), and other factors like biomass capacity and biomass growth rate that are affected by external factors. Equation 1 formalizes these relationships by providing an analytical expression for the minimum speed of the lysis wavefront as a function of phage properties and cell morphology.

Then, in Equation 2, the relative squared speed allowed cancelling out many constants thus allowing the model to be compared with the experimental data. This theoretical formulation aligns well with experimental data. As shown in Fig 5a, mecillinam treatment, which increases cell width without affecting length, produces a linear relationship between plaque speed and width. Conversely, ciprofloxacin induced filamentation (increased length without altering width), and the model predicts a non-linear relationship with an optimal length for plaque expansion (Fig 5b). The fits to the experimental data demonstrate high agreement, which is linear for mecillinam and quadratic for ciprofloxacin, validating the predictive capacity of the model. From our theoretical model emerges an optimal bacterial length that maximizes the terminal speed under filamentation conditions. This optimal length ($l_{opt}=\beta /\text{2}\gamma$) relates to the replication rate per unit length γ and the release rate β. This result arises directly from the model and is not immediately evident from raw experimental observations, underscoring the value of formalizing the dynamics.

Furthermore, the model generalizes beyond the two specific antibiotics tested. It provides a conceptual and quantitative framework for understanding how any antibiotic that modifies bacterial morphology could influence cell lysis efficiency. Additionally, it suggests that other parameters, such as phage adsorption rates (as shown in S4 Fig), can significantly modulate plaque expansion depending on host physiological state.

Interestingly, the model also aligns with known biological mechanisms regulating cell shape. Morphological parameters like length and width correspond to the rate of division protein (FtsZ) accumulation and cell wall synthesis via MreB, respectively, as described by Ojkic et al. [33]. Ciprofloxacin-induced SOS response inhibits FtsZ assembly through SulA production leading to filamentation [34], while mecillinam inhibits PBP2, disrupting MreB function [35], thus increasing cell width. These molecular changes map directly to the morphological parameters in our model, offering a mechanistic link between antibiotic action and phage propagation dynamics.

Limitations and future directions: while the model captures key trends, it is important to acknowledge its simplifications. To retain analytical tractability, we assumed:

- Phage adsorption and release are fast relative to diffusion.
- The biomass encountered by the traveling wave changes slowly compared to wavefront speed.
- The latent period is not explicitly modeled, though its effects may be indirectly represented through parameters such as the average replication rate (γ) and release rate (β). For example, a large β and small γ can phenomenologically reflect a longer latent period.

Despite these simplifications, the model offers a useful and intuitive representation of phage-bacteria dynamics. It complements the experimental results and extends to potentially estimating the speed of lysis front for different morphological parameters.

Together, our theoretical framework and experimental data reveal a key impact of cell morphology on phage epidemics in structured environments. In a previous work, we investigated the effect of cell elongation in liquid and well-agitated cultures [13]. However, most natural environments are far from being so homogenous and an increasing number of phage-host interaction studies focuses on more complex matrices such as gut or soil [36,37]. As for phage therapy, whatever the body parts where phages are administered, it is likely that the environment will be structured and much less homogenous than an agitated flask. This is why we moved to semi-solid medium, to start to replace phage and antibiotics interactions in a physical context a little closer to reality.

## Materials and methods

### Bacterial strains, phages and culture conditions

Bacteriophage T5 was kindly provided by P. Boulanger’s team and bacteriophage T7 was obtained from the DSMZ collection. Bacterial host *E. coli* MG1655 used in this study originated from P. Genevaux’s collection. *E. coli* LC-E75 strain carrying a chromosomal copy of *dcas9* gene under the *P*_tet_ promoter was provided by David Bikard (Addgene #115925). *dcas9* encodes a *Streptoccocus pyogenes* Cas9 mutant inactivated in its endonuclease function [38]. Liquid cultures were carried out at 37°C, 180 rpm in Lysogeny Broth (Thermo Fisher). For solid and semisolid media, 1.5% or 0.5% agar (Sigma-Aldrich) were added, respectively. Antibiotics mecillinam (Sigma-Aldrich), ciprofloxacin (Merk), ceftazidime (Sigma-Aldrich), kanamycin (Sigma-Aldrich) 50 µg/mL and chloramphenicol (Sigma-Aldrich) 25 µg/mL were added at the indicated concentrations. Anhydrotetracycline (aTc Sigma-Aldrich) was used at the indicated concentrations to induce *dCas9* gene expression.

### Plasmids and sgRNA spacers

Plasmid psgRNA coding for a single-guide RNA (sgRNA) used by dCas9 was a gift from David Bikard (Addgene plasmid # 11400). *mreB-* an *ftsZ-* targeting sgRNA were designed by hybridizing complementary primers carrying the following spacers: psgRNA-*ftsZ* 5’-CCTGAGGCCGTAATCATCGT-3’ and psgRNA-*mreB* 5’-GATATCAACCACCATAGAAC- 3’. These spacers were flanked by two BsaI restriction sites and inserted into the psgRNA through Golden Gate assembly (NEB), thus generating *ftsZ*-sgRNA and *mreB*-sgRNA. The non-targeting sgRNA (sgRNA) refers to the unmodified plasmid. All three plasmids were transformed into chimiocompetent *E. coli* LC-E75 cells.

### Determination of antibiotics and inducer concentrations

In order to determine for each antibiotic the maximum concentration on plates that does not perturbs bacterial growth, we plated a well-mixed, log-phase bacterial culture in a 90 mm petri dish as follows: a volume of 5 mL of soft-agar (0.5%) containing the bacterial culture at an initial optical density measured at 600 nm (OD_600_) of 0.1 was poured on top of a 20 mL layer of hard-agar (1.5%) devoid from bacterium. Antibiotics were diluted in the hard-agar layer and the indicated concentrations were given for the total solid medium volume (5 mL soft-agar + 20 mL hard-agar). Plates were then incubated overnight at 37°C. The bacterial lawn aspect was used to determine the maximum antibiotic concentration that neither significantly impacted the opacity of the bacterial lawn nor produced grainy or heterogenous textures on the Petri dish surface. These sub-inhibitory concentrations were set at 15 ng/mL for ciprofloxacin, 120 ng/mL for ceftazidime, 200 ng/mL for mecillinam, 3 µg/mL for chloramphenicol and 2 µg/mL for kanamycin. *E. coli* cell rounding or filamentation was reproduced without antibiotics by repressing *mreB* and *ftsZ* gene expression, respectively. aTc was used to induce *dCas9* gene expression and the maximum concentration set at the value leading to morphological changes without eliciting significant cell death.

### Lysis plaque radii measurements

Phage lysis plaque expansion was monitored on plates by recording images with a stereomicroscope equipped with a 1X objective (Nikon SMZ800N). For single time point analysis, phage lysis plaques were left to propagate for 24 hours at 37°C then a single image was taken. For T7 plaque formation kinetics, we followed three distinct lysis plaques on a plate incubated at 37°C with image acquisition every 15 minutes. Plaque radii were determined using the ImageJ plugin “Radial Profile Angle” (Paul Baggethun, Pittsburgh, PA) and selecting a radius of 1000 pixels (8.62 mm) centered on each lysis plaque. This plugin averages, over 360°, the pixel intensity along the plaque radius, starting from the center of the plaque, resulting in a sigmoid curve with the inflection point at the edge of the plaque. Since the pixel value is averaged over 360°, the presence of an overlapping plaque will not impair the measurement. The resulting averaged intensity profiles were normalized using the formula $I_{normalized}=\frac{(I-I_{min)}}{\left(I_{max}-I_{min}\right)}$. After normalization, a sigmoid function was fitted to each measured plaque intensity profile using the non-linear least squares function from the R software and the following formula $:\;y=a\frac{L}{\text{1}+e^{-k(x-x_{\text{0}})}}$ (where a and L are the lower and upper asymptotes, respectively, k the growth rate or “steepness” of the curve, and $x_{\text{0}}$ as the x value of the function midpoint). The radius value for a given plaque was considered to be the inflection point of the fitted sigmoid curve.

### Assessing antibiotics’ effect on bacterial morphology

A log-phase *E. coli* MG1655 culture was diluted to a final OD_600_ of 0.025 in 10 mL of LB medium, with or without addition of subinhibitory concentrations of antibiotics. After 2 hours incubation at 37°C under agitation at 180 rpm, a culture sample (OD_600_ ≈ 0.8) was fixed by diluting 1:1 in PBS buffer PFA 4% solution. Bacterial cells were imaged on an inverted phase-contrast microscope (Nikon TiE) using an oil immersion 100X NA 1.45 objective and Nikon’s NIS-Element software. Large fields were captured to ensure statistical meaning of the analyzed population.

### Image analysis and morphological changes determination

Cell image analysis was performed using MicrobeJ [39] after treatment with Omnipose, a bacterial segmentation tool [40]. From the output mask we then measured total length (*l*) from pole to pole and width (*w*) for each individual cell. To estimate bacterial surface area (*S*) and volume (*V*), each cell was modelled as a cylinder (length *l* – *w* and width *w*) capped with two hemispheres (diameter *w*) on each extremity:


S=𝛱*w*l

$$V=\frac{𝛱}{\text{4}}*w^{\text{2}}(l-\frac{w}{\text{3}})\;$$

Statistical analysis was conducted in R [41] and figures were produced using the ggplot2 package [42].

### T5 and T7 latent period and burst size determination

Latent period (time between genome injection and release of the first virion by cell lysis) and burst size (number of virions produced per infected cell) were determined in liquid medium through the One-Step Growth Curve experiment [23]. Log-phase cultures of *E. coli* LC-E75, carrying sgRNAs targeting either *mreB*, *ftsZ* or nothing, were infected at low Multiplicity Of Infection (MOI ~ 0.001) at 37°C and 180 rpm. In all cases, induction of dCas9 took place 2 hours prior to phage infection in order to allow the dCas9/sgRNA-mediated morphological change to take place. Five min post-infection, 100 µL culture samples were then regularly assayed (every 5.0 and 2.5 min for T5 and T7, respectively) and plated using the top-agar overlay method with *E. coli* MG1655 as indicator strain. The assessment of morphological changes induced by dCas9 expression was confirmed by microscopy on culture aliquots sampled shortly before infection. After overnight incubation of the plated samples, the number of Plaques Forming Units (PFU) at each timepoint was calculated and plotted against time.

### Experimental data fitting

In order to determine T7 lysis plaque expansion terminal velocities, experimental $(r-r_{\text{0}}$kinetics for each condition displayed in S1 Fig were fitted with a linear regression $y=\;a_{\text{1}}.x+a_{\text{0}}$ in the 15 – 20 h time range (terminal velocity c is equal to $a_{\text{1}}$). Both $a_{\text{0}}\;$and $a_{\text{1}}$ coefficients were computed according to the weighted least squares regression method. Since for each condition tested $(r-r_{\text{0}}$curve is the mean of three kinetics recorded on three individual lysis plaque, the standard deviation of the experimental data was used to calculate the terminal velocity c standard deviation.

The experimental squared relative terminal velocities with respect to cell width w (Fig 5a)or cell length l (Fig 5b) was fitted with a linear regression $y=\;a_{\text{1}}.x+a_{\text{0}}$ (Fig 5a) or a 2^nd^ degree polynomial function $y=\;a_{\text{2}}.x^{\text{2}}+a_{\text{1}}.x$ (Fig 5b) in accordance with our theoretical model also using the weighted least squares regression method. The standard deviation of each $a_{i}$ parameter was computed using the terminal velocities and morphological parameters standard deviations.

The weighted least squares regression method was implemented in Excel and all calculations can be found in S1 Table.

## Supporting information

S1 Fig Bacterial count in the presence of antibiotics that do not alter cell morphology. Mean number of normalized bacterial count within *E. coli* microcolonies under maximal subinhibitory chloramphenicol and kanamycin concentrations. *N* = between 16–18 microcolonies per condition were imaged, starting from one or few *E. coli* individuals over 105 min of growth at 37°C in the presence or absence of the antibiotics. Error bars represent the standard error of the mean (S.E.M.).
(TIFF)

S2 Fig Phage T5 and T7 plaque radii *vs* bacterial morphological changes. Comparison of phage T5 and T7 plaque radii in the presence of *E. coli* morphological changes induced by antibiotics or host gene repression. **Panels a-f: cell morphological changes induced by sublethal doses of antibiotics.** Lysis plaque radii in the presence of ciprofloxacin inducing cell filamentation (**panels a** and **b** for T5 and T7, respectively), in the presence of ceftazidime inducing cell filamentation (**panels c** and **d** for T5 and T7, respectively) and in the presence of mecillinam inducing cell bloating (**panels e** and **f** for T5 and T7, respectively). The graphs highlight the principal morphological changes in plaque formation induced by each antibiotic. **Panels g-j: cell morphological changes induced by dCas9-induced host gene repression**. Lysis plaques radii during *mreB* inhibition inducing cell bloating (**panels g** and **i** for T5 and T7, respectively) and during *ftsZ* inhibition inducing cell filamentation (**panels h** and **j** for T5 and T7, respectively). For each condition, between *N *= 18–414 lysis plaques as well as *N *= 171–2,684 individual bacteria were measured. Whiskers represent the standard error of the mean (S.E.M.).
(TIFF)

S3 Fig Propagation profiles of phage T7 epidemics under PAS conditions. Radius increase of T7 lysis plaques between 3 and 28 hours post infection in the presence of increasing concentration of mecillinam (a) or ciprofloxacin (b). *N* = at least 3 independent lysis plaques were recorded per condition. The shadowed areas represent the standard error of the mean of each curve (S.E.M.).
(TIFF)

S4 Fig Effect of model parameters on phage lysis plaque expansion. Phage lysis plaque radius r expansion kinetics (main panels) are obtained by integration of velocity changes over time (insets). The velocity scaling factor δβ is kept constant at value 1.0 (Eq. 18). **(a, b)** *Effect of cell morphological parameters.* Changing cell length l (**a**) and cell width w (**b**). **(c, d)** *Effect of phage parameters.* Changing phage adsorption (**c**) and phage inactivation (**d**). **(e, f)** *Effect of external parameters.* Changing biomass growth rate (**e**) and biomass capacity Vmax (**f**).
(PNG)

S1 Text Modeling of T7 lysis plaque propagation. The text includes detailed equations corresponding to the mathematical model.
(DOCX)

S1 Table Raw data and statistics. Raw data and statistics used to build the main and supplemental figures are included in the table.
(XLSX)
