# Supplementary figures and images for: Antibiotic-induced morphological changes enhance phage predation
Source: PLoS Pathog. 2025 Oct 3;21(10):e1013546. doi: 10.1371/journal.ppat.1013546 (PMC12510666; doi:10.1371/journal.ppat.1013546)

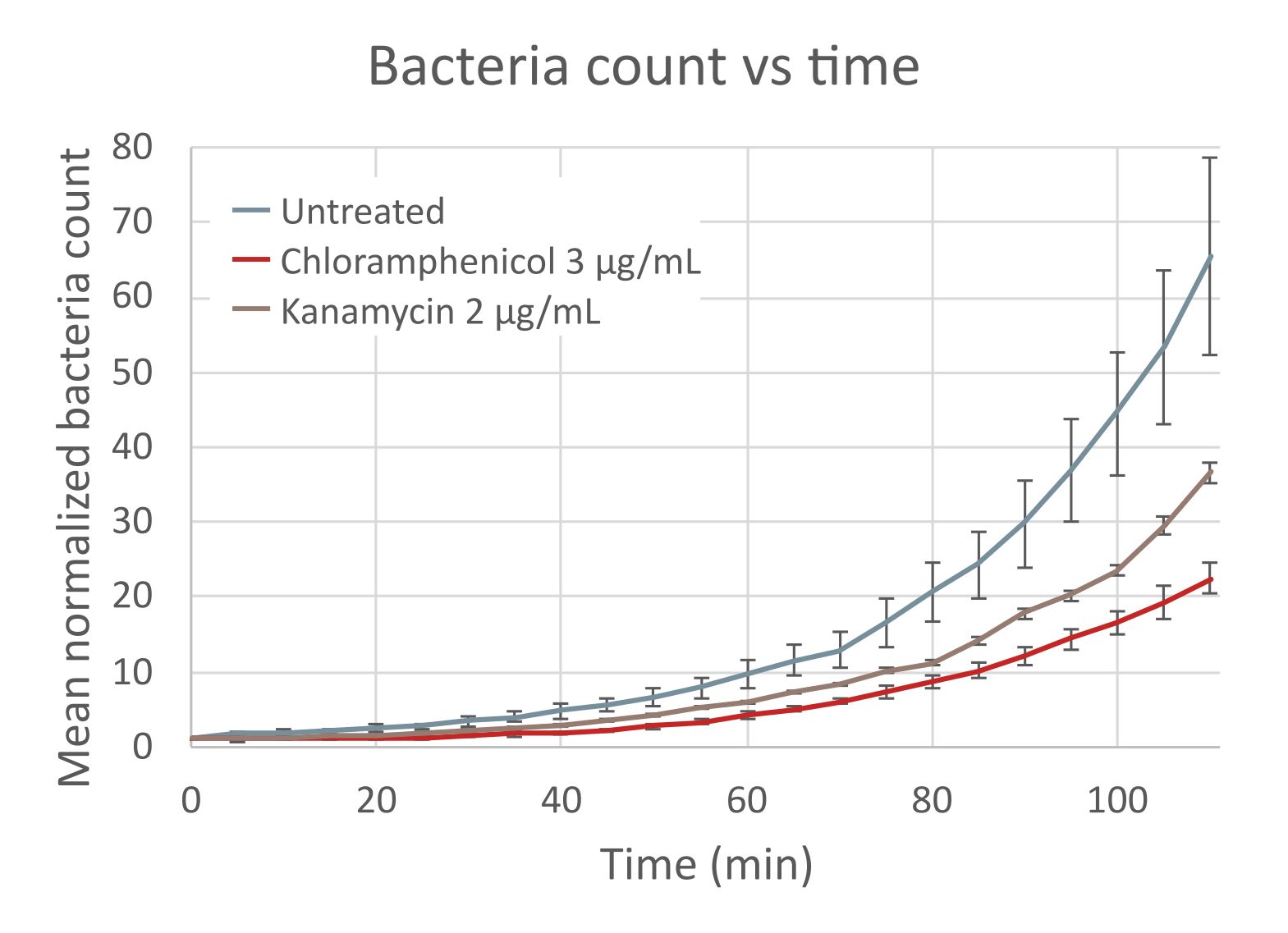

Supplement: S1 Fig — Mean number of normalized bacterial count within E. coli microcolonies under maximal subinhibitory chloramphenicol and kanamycin concentrations. N = between 16–18 microcolonies per condition were imaged, starting from one or few E. coli individuals over 105 min of growth at 37°C in the presence or absence of the antibiotics. Error bars represent the standard error of the mean (S.E.M.). (TIFF) [file ppat.1013546.s001.tiff]

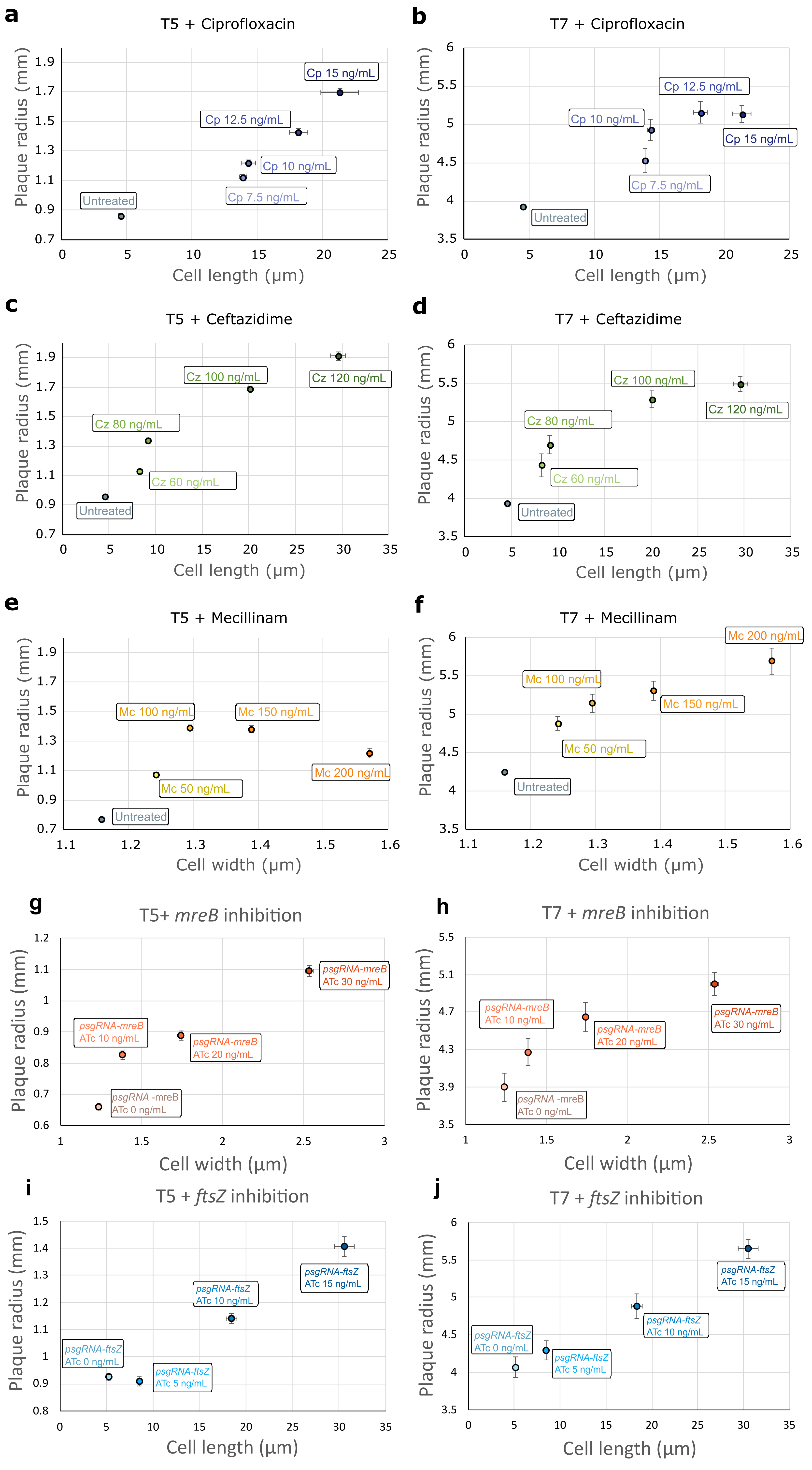

Supplement: S2 Fig — Comparison of phage T5 and T7 plaque radii in the presence of E. coli morphological changes induced by antibiotics or host gene repression. Panels a-f: cell morphological changes induced by sublethal doses of antibiotics. Lysis plaque radii in the presence of ciprofloxacin inducing cell filamentation (panels a and b for T5 and T7, respectively), in the presence of ceftazidime inducing cell filamentation (panels c and d for T5 and T7, respectively) and in the presence of mecillinam inducing cell bloating (panels e and f for T5 and T7, respectively). The graphs highlight the principal morphological changes in plaque formation induced by each antibiotic. Panels g-j: cell morphological changes induced by dCas9-induced host gene repression. Lysis plaques radii during mreB inhibition inducing cell bloating (panels g and i for T5 and T7, respectively) and during ftsZ inhibition inducing cell filamentation (panels h and j for T5 and T7, respectively). For each condition, between N = 18–414 lysis plaques as well as N = 171–2,684 individual bacteria were measured. Whiskers represent the standard error of the mean (S.E.M.). (TIFF) [file ppat.1013546.s002.tiff]

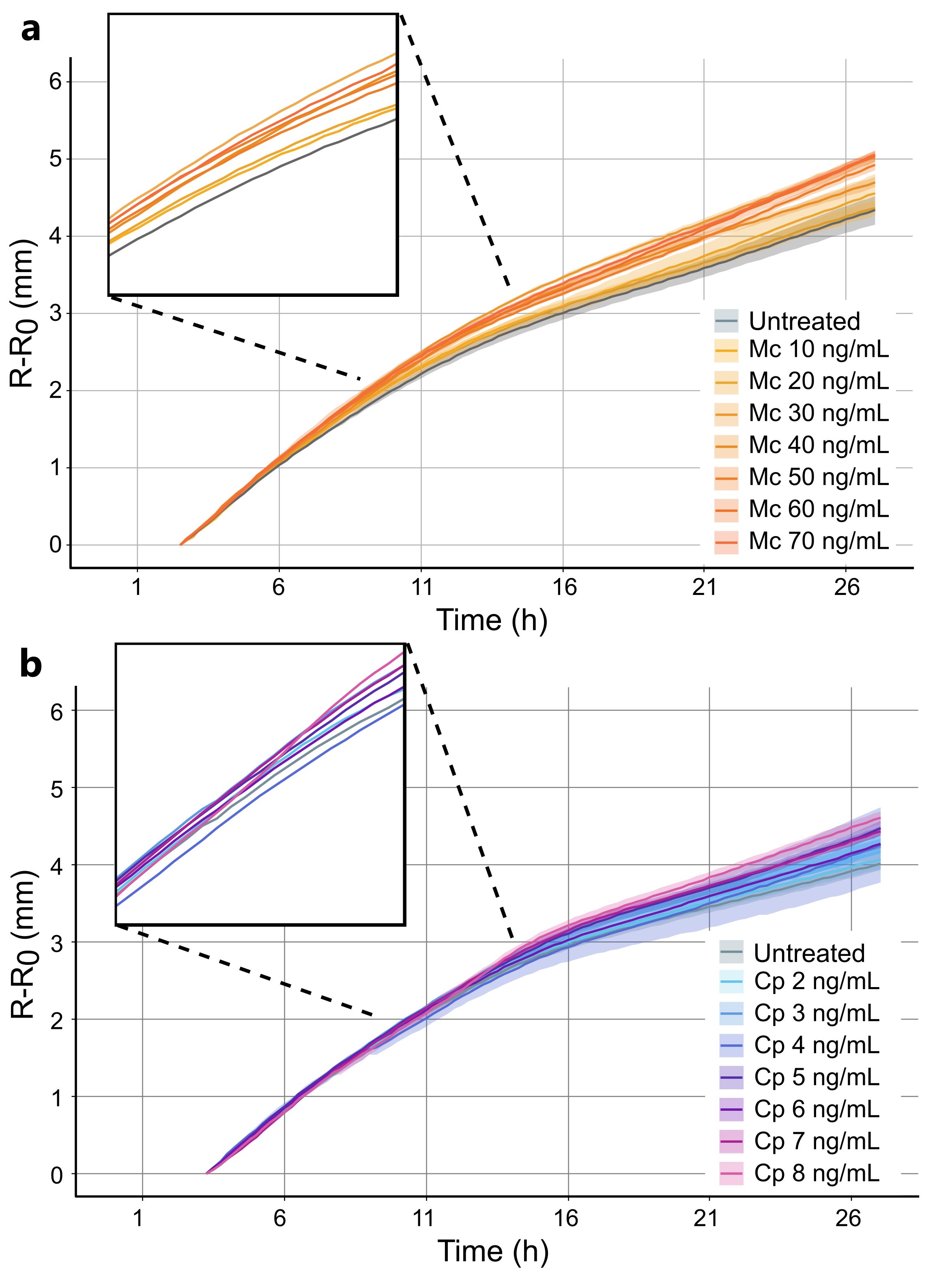

Supplement: S3 Fig — Radius increase of T7 lysis plaques between 3 and 28 hours post infection in the presence of increasing concentration of mecillinam (a) or ciprofloxacin (b). N = at least 3 independent lysis plaques were recorded per condition. The shadowed areas represent the standard error of the mean of each curve (S.E.M.). (TIFF) [file ppat.1013546.s003.tiff]

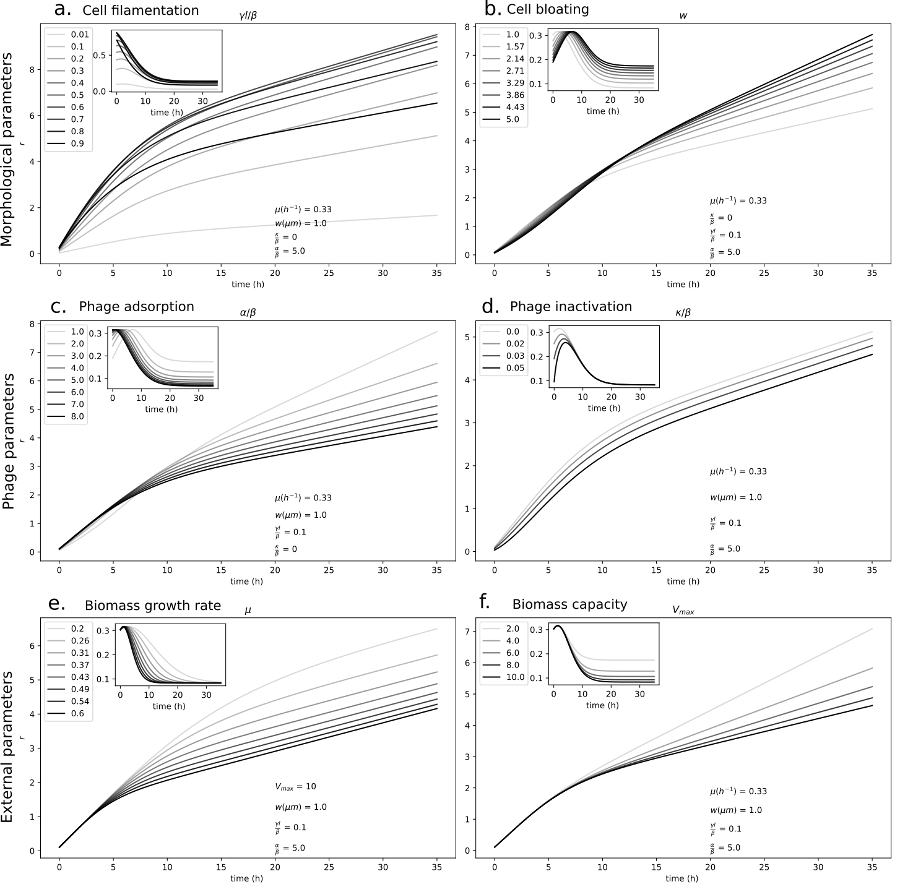

Supplement: S4 Fig — Phage lysis plaque radius r expansion kinetics (main panels) are obtained by integration of velocity changes over time (insets). The velocity scaling factor δβ is kept constant at value 1.0 (Eq. 18). (a, b) Effect of cell morphological parameters. Changing cell length l (a) and cell width w (b). (c, d) Effect of phage parameters. Changing phage adsorption (c) and phage inactivation (d). (e, f) Effect of external parameters. Changing biomass growth rate (e) and biomass capacity Vmax (f). (PNG) [file ppat.1013546.s004.png]
