# Supplementary material for: Antibiotic-induced morphological changes enhance phage predation
Source: PLoS Pathog. 2025 Oct 3;21(10):e1013546. doi: 10.1371/journal.ppat.1013546 (PMC12510666; doi:10.1371/journal.ppat.1013546)
Supplement: S1 Text — The text includes detailed equations corresponding to the mathematical model. (DOCX) [file ppat.1013546.s005.docx]

**Supporting Text for**

**Antibiotic-induced morphological changes boost phage propagation through diverse mechanisms**

Julián Bulssico^1,*^, Swapnesh Panigrahi^1^, Mélanie Matveeva^1^, Nicolas Ginet^1^, and Mireille Ansaldi^1,*^

^1^Laboratoire de Chimie Bactérienne, UMR7283, Institut de Microbiologie de la Méditerranée, Centre National de la Recherche Scientifique, Aix-Marseille Université, Marseille, France

* Corresponding authors: [julianbulssico@gmail.com](mailto:julianbulssico@gmail.com); [mireille.ansaldi@cnrs.fr](about:blank)

**This PDF file includes:**

Modeling of T7 lysis plaque propagation, including detailed equations corresponding to the mathematical model

**Modeling of T7 lysis plaque propagation**

*Parameters*

*F* Free phages fraction

*A* Adsorbed phages fraction

*P* Total phage concentration

*d* Phages intrinsic diffusion coefficient (µm^2^.min^-1^)

*k* Phage inactivation rate (min^-1^)

*µ* Biomass growth rate (h^-1^)

*V* Biomass volume (µm^3^)

$l$ Cell length (µm)

$w$ Cell width (µm)

$s$ Cell surface area (µm^2^)

$v$ Cell volume (µm^3^)

*s* surface area-to-volume ratio (µm^-1^)

*a* Phage adsorption rate (cell^-1^.µm^-2^.min^-1^)

*g* Phage replication rate per unit cell length (min^-1^.µm^-1^)

*b* Phage release rate (min^-1^)

*Equations*

In our system, the total phage concentration $P$ dynamics is a function of the dynamics of two “species” of phages within the sample, namely, “free” phages (denoted by $F$) and “absorbed” phages (denoted by *A*). “Free” phages diffuse freely through the substrate with an intrinsic diffusion coefficient of $\delta$ ($\mu m^{2}min^{-1}$) and become inactive at a rate $\kappa\left( min^{-1} \right)$. In the presence of *E. coli* susceptible cells, free phages adsorb onto the cell surface with a rate proportional to the cell surface area (*s*) (10). We consider the biomass volume *V* and the cell volume $v$to write the rate of adsorption as $\alpha\frac{s}{v}V$, with an adsorption rate per surface area per cell $\alpha$ ($cell^{-1}\mu m^{-2}min^{-1})$. We assumed adsorbed phages replicate within the cell with a rate proportional to the cell length ($l$) and rate per unit length $\gamma$ ($min^{-1}\mu m^{-1}$) (1). Phages are released from the cell at a constant rate $\beta$ ($min^{-1}$). Then, the dynamical equations for the free and adsorbed phages are written as:

$F_{t}=\delta F_{xx}-\alpha\frac{s}{v}VF+\beta A-\kappa F$ (Eq. 1)

$A_{t}=\alpha\frac{s}{v}VF+\gamma lA-\beta A$ (Eq. 2)

$P_{t}=F_{t}+ A_{t}$ (Eq. 3)

where the subscripts *t* and *xx* represent the derivative with respect to time, and second derivative in space, respectively. Under the assumption that the diffusion is slower than the adsorption and release of the phages, then at steady state, $A_{t}\to0$. Noting that $P=F+A$ we write,

$A=\frac{\lambda\sigma V}{1+\lambda\sigma V}P$ (Eq. 4)

$F=\frac{1}{1+\lambda\sigma V}P$ (Eq. 5)

where $\lambda=\frac{\alpha/\beta}{1-\gamma l/\beta}$. Inserting Eq. 4 and 5 in Eq. 1 and 2 we write the dynamical equation for the total phage concentration under the assumption that the biomass volume $V$ grows slowly compared to the above dynamic equations, we simplify the total phage concentration $P$ as:

$P_{t}=\frac{\delta}{1+\sigma\lambda V}P_{xx}+\gamma l\frac{\sigma\lambda V}{1+\sigma\lambda V}P-\frac{\kappa}{1+\lambda\sigma V}P$ (Eq. 6)

where $\sigma=s/v$. Then, we look for a travelling wave front solution of the form $P\left( z \right)=P\left( x-ct \right)$. We thus obtain:

$-c\left[ P\left( z \right) \right]_{z}=\frac{\delta}{1+\sigma\lambda V}P\left( z \right)_{zz}+\gamma l\frac{\sigma\lambda V}{1+\sigma\lambda V}P\left( z \right)-\frac{\kappa}{1+\lambda\sigma V}P\left( z \right)$ (Eq. 7)

$P\left( z \right)_{z}=Q\left( z \right)$(Eq. 8a)

$Q\left( z \right)_{z}=-\frac{c}{\delta}\left( 1+\sigma\lambda V \right)Q\left( z \right)-\gamma l\sigma\lambda VP\left( z \right)/\delta+\kappa P\left( z \right)/\delta$ (Eq. 8 )

The Jacobian *J* near steady state is then:

$J=$ $\left[ {}_{\frac{\kappa}{\delta}-\frac{\gamma\lambda l\sigma V}{\delta}}^{0}{}_{-\frac{c\left( \lambda\sigma V+1 \right)}{\delta}}^{1} \right]$ (Eq. 9)

with eigenvalues *eig*:

$eig=-\frac{c\left( \lambda\sigma V+1 \right)\pm\sqrt{\left( c\lambda\sigma V+c \right)^{2}+4\delta\left( \kappa-\gamma\lambda l\sigma V \right)}}{2\delta}$ (Eq. 10)

Real roots exist under the condition that:

$c^{2}\geq4\delta\frac{\gamma l\lambda\sigma V-\kappa}{\left( 1+\lambda\sigma V \right)^{2}}$ (Eq. 11)

leading to the expression for the minimum speed of the travelling wavefront solution. In a model where a bacterium is represented by a cylinder caped by two hemispheres, cell surface area $s$, cell volume $v$ and the surface area-to-volume ratio s are:

$s = \pi w \left( l - w \right)+ 4 \pi\left( \frac{w}{2} \right)^{2}= \pi l w$ (Eq. 12)

$v = \pi\left( \frac{w}{2} \right)^{2} \left( l - w \right)+ \frac{4}{3} \pi\left( \frac{w}{2} \right)^{3}=\frac{4}{3} \pi lw^{2}-\frac{1}{12} \pi w^{3}$ (Eq. 13)

$\sigma=\frac{s}{v}= 12 \frac{l}{w \left( 3l - w \right)}\sim\frac{const.}{w}$ (for large $l$) (Eq. 14)

Since we modelled a bacterium as a cylinder caped by two hemispheres, when the length is large as in the case of filamentous cells the surface area-to-volume ratio is inverse of the width of bacteria, *i.e.*, $\sigma\sim1/w$. The stability analysis of this solution to travelling wavefront solution of the form $P\left( x-ct \right)$ yields the minimum speed of the wavefront $\left| c \right|$ in the form of:

$\left| c \right|\geq2\sqrt{\delta\beta}\frac{\sqrt{\frac{\gamma l}{\beta}\lambda\sigma V-\frac{\kappa}{\beta}}}{1+\lambda\sigma V}$ (Eq. 15)

The solution thus exists under the conditions that replication rate is slow, $\gamma l<\beta$ and the virion inactivation rate is small, $\kappa<\gamma l\lambda\sigma V$. The speed is shown to be a function of a characteristic speed $\sqrt{\delta\beta}$, morphological parameters $\frac{\gamma l}{\beta},\sigma$, phage-related parameter $\alpha/\beta$ and the biomass volume $V$ encountered by the travelling wave. We thus defined from Eq. 4 the dimensionless speed $c/\sqrt{\delta\beta}$ as:

$c/\sqrt{\delta\beta}=2\frac{\sqrt{\frac{\gamma l}{\beta}\lambda\sigma V-\frac{\kappa}{\beta}}}{1+\lambda\sigma V}$ (Eq. 16)

Under the conditions that the biomass encountered by the wavefront changes relatively slower than the speed of the lysis wavefront and by modelling the biomass in-front of the traveling wave as a logistic growth with growth rate $\mu$ we can obtain a time-dependent speed by defining $V\left( t \right)$:

$V\left( t \right)=\frac{V_{max}}{1+\left( \frac{V_{max}}{V_{0}}-1 \right)e^{-\mu t}}$ (Eq. 17)

where $V_{max}$ is the carrying capacity and $V_{0}$ is the initial biomass volume.

In the case where phage deactivation is negligible ($\kappa\ll\gamma l$), which seems to be the case for T7 phage, the speed of the travelling wavefront $\left| c \right|$ (Eq. 15) can be simplified as:

$\left| c \right|\geq2\frac{\sqrt{\delta\beta} \sqrt{\frac{\gamma l}{\beta}\lambda\sigma V}}{1+\lambda\sigma V}$ (Eq. 18)

Furthermore, when the host reaches biomass capacity $V_{max}$ (and assuming $\lambda\sigma V_{max}\gg1$), the travelling wavefront reaches a terminal velocity $c$:

$c\sim2\sqrt{\frac{\delta\gamma l}{\lambda\sigma V_{max}}}\sim2\sqrt{\frac{\delta\gamma}{V_{max}}} \sqrt{\frac{lw}{\lambda}}$ (Eq. 19)

Thus, the square of speed in each experimental case $c_{T}$ (*T* for antibiotic-treated cells) relative to the speed of untreated host $c_{u}$ (*u* for untreated cells) is:

$\left( \frac{c_{T}}{c_{u}} \right)^{2}=\frac{V_{max,u}}{V_{max,T}}\frac{l_{T}w_{T}}{l_{u}w_{u}}\frac{1-\frac{\gamma l_{T}}{\beta}}{1-\frac{\gamma l_{u}}{\beta}}$ $\left( \frac{{}_{T}}{{}_{u}} \right)^{2} \frac{{}_{T}{}_{T}}{{}_{u}{}_{u}}\frac{\frac{{}_{T}}{\beta}}{1-\frac{{}_{u}}{\beta}}$ (Eq. 20)

When cell length $l$ is constant (*e.g.*, with mecillinam), Eq. 20 can be simplified as a first-degree polynomial equation with a null intercept:

$\left( \frac{c_{T}}{c_{u}} \right)^{2}=\frac{w_{T}}{w_{u}}=a_{1}*w_{T}$ (Eq. 21)

with $a_{1}= \frac{1}{w_{u}}\frac{V_{max,u}}{V_{max,T}}$.

When cell width $w$ is constant (*e.g.*, with ciprofloxacin), Eq. 18 can be simplified as a second-degree polynomial equation with a null intercept:

$\left( \frac{c_{T}}{c_{u}} \right)^{2}=\frac{V_{max,u}}{V_{max,T}}\frac{1}{l_{u}(1-\frac{\gamma}{\beta}l_{u})}l_{T}*\left( 1-\frac{\gamma}{\beta}l_{T} \right)=b_{1}*l_{T}*(1-b_{2}*l_{T})$ (Eq. 22)

with $b_{1}=\frac{V_{max,u}}{V_{max,T}} \frac{1}{l_{u}(1-\frac{\gamma}{\beta}l_{u})}$ and $b_{2}= \frac{\gamma}{\beta}$.

**Reference**

1. S. Kannoly, *et al.*, Single-Cell Approach Reveals Intercellular Heterogeneity in Phage-Producing Capacities. *Microbiology Spectrum* **11**, e02663-21 (2022).
